# Exploring the link: magnesium intake and hepatic steatosis in Americans

**DOI:** 10.3389/fnut.2024.1367174

**Published:** 2024-05-23

**Authors:** Xingxing Chen, Liying Fu, Zhongxin Zhu, Yunchao Wang

**Affiliations:** ^1^Clinical Research Center, Xiaoshan Affiliated Hospital of Wenzhou Medical University, Hangzhou, Zhejiang, China; ^2^Voluntary Blood Donation Service Center of Xiaoshan District, Hangzhou, Zhejiang, China; ^3^Department of General Practice, The First People's Hospital of Xiaoshan District, Hangzhou, Zhejiang, China

**Keywords:** magnesium, hepatic steatosis, fatty liver, controlled attenuation parameter, NHANES

## Abstract

**Purpose:**

The connection between magnesium and hepatic steatosis has not been well-studied. This study aimed to explore the link between magnesium intake and hepatic steatosis, utilizing data from the National Health and Nutrition Examination Survey (NHANES) 2017–2020.

**Materials and methods:**

The analysis included 5,935 participants, excluding individuals with hepatitis infection or substantial alcohol consumption. Magnesium intake assessment was based on 24-h dietary recalls. Hepatic steatosis evaluation employed the controlled attenuation parameter (CAP), measured via transient elastography. Multivariate regression and subgroup analyses were conducted to scrutinize the relationship between magnesium intake and CAP values.

**Results:**

A higher magnesium intake was associated with lower CAP values, after adjusting for potential confounders. Subgroup analyses indicated an inverted U-shaped correlation between magnesium intake and CAP in women, White people, and non-hypertensive individuals, with respective inflection points at 126, 124.5, and 125 mg/day, respectively. Below these thresholds, a higher magnesium intake correlated with increased CAP values, while above these points, it was associated with decreased CAP.

**Conclusion:**

This extensive population-based study indicates an inverse relationship between magnesium intake and hepatic steatosis in Americans. This relationship displays an inverted U-curve, varying before and after specified inflection points in women, White people, and non-hypertensive individuals. These findings offer insights into tailored magnesium supplementation strategies for preventing and treating liver steatosis, based on gender and ethnicity.

## Introduction

Hepatic steatosis occurs when fat droplets accumulate within liver cells (1). Fatty liver is diagnosed when the liver's fat content exceeds 5% of its weight (2). The severity of fatty liver varies, ranging from simple fatty liver, which shows no evident inflammation or damage, to non-alcoholic steatohepatitis (NASH), characterized by significant inflammation and liver cell damage (3). The global prevalence of fatty liver is rising in parallel with the obesity epidemic, affecting an estimated 25% of the global population (4). While simple fatty liver can often be managed with lifestyle and dietary changes, about 30% of cases may progress to NASH (5). Without treatment, it can progress to liver cirrhosis, liver cancer, or liver failure (6). Therefore, early diagnosis and prevention of liver steatosis are crucial (7).

Magnesium, a vital mineral, is involved in more than 300 enzymatic reactions in the human body, such as energy production, carbohydrate metabolism, protein synthesis, and regulating blood pressure (8). Serving as a cofactor for numerous enzymes in carbohydrate and lipid metabolism, magnesium plays a crucial role in metabolic processes (9). Preliminary research links low magnesium intake with a heightened risk of metabolic diseases like type 2 diabetes and cardiovascular disorders (10, 11).

The link between magnesium and fatty liver disease is currently under active investigation. Limited studies have explored the role of serum or dietary magnesium in metabolic dysfunction-associated steatotic liver disease (MASLD) (12–14). Nevertheless, hepatic steatosis in the general population is not well-studied in these investigations. Additionally, previous studies have predominantly diagnosed MASLD using abdominal ultrasonography, a method significantly influenced by the physician's subjective judgment and technical expertise.

The Controlled Attenuation Parameter (CAP), assessed semi-automatically via liver elastography, offers a reliable, non-invasive approach to quantitatively evaluate hepatic steatosis (15). Many studies, corroborated by liver biopsy, demonstrate a significant correlation between CAP values and liver fat levels (16–18). Between 2017 and 2020, the National Health and Nutrition Examination Survey (NHANES) introduced transient elastography to measure one hepatic steatosis, yielding the largest dataset of CAP observations in the United States. Herein, our study aims to examine the relationship between magnesium intake and hepatic steatosis by analyzing extensive data on magnesium intake and CAP values from the 2017–2020 NHANES. We will particularly focus on whether there are unique associations across different demographic subgroups.

## Materials and methods

### Statement of ethics

This study received approval from the National Center for Health Statistics Research Ethics Review Board, with each participant providing consent.

### Study population

**Figure 1:**
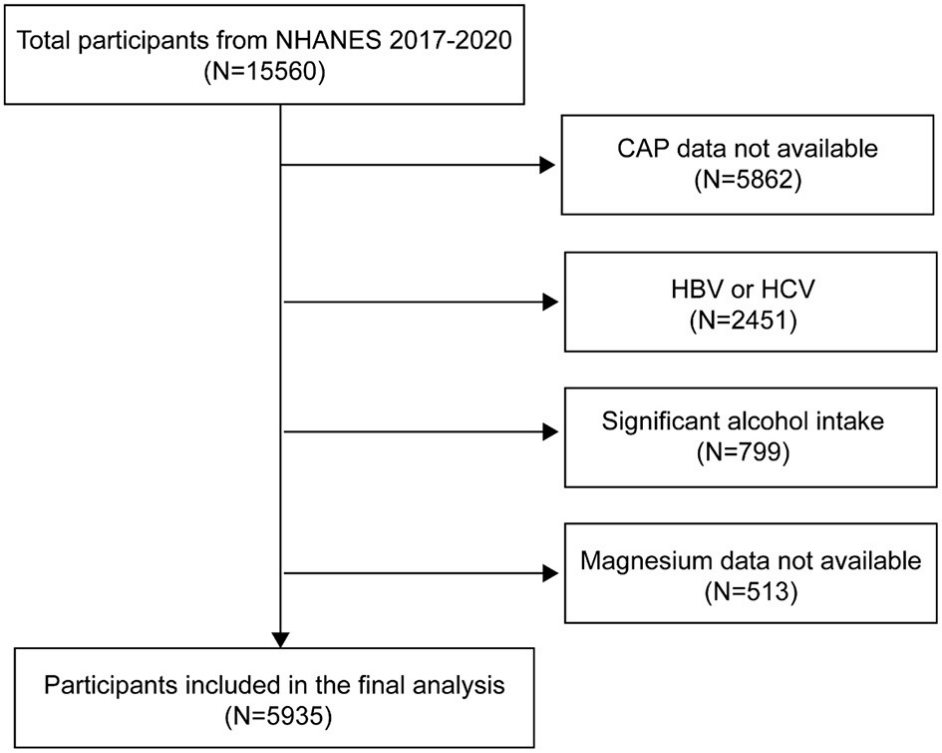
Flowchart of participant selection. NHANES, National Health and Nutrition Examination Survey; CAP, controlled attenuation parameter.

To ensure nationwide representation, NHANES, an extensive, continuous cross-sectional survey in the US, employs stratified, multistage, clustered random sampling to gather diet and health data from the entire population (19). Of the 15,560 participants in the 2017–2020 NHANES cycle, 9,698 had available CAP data. We excluded 2,451 participants who tested positive for hepatitis B antigen, hepatitis C antibody, or hepatitis C RNA, 799 with significant alcohol consumption (four or more drinks daily), and 513 lacking magnesium intake data. Ultimately, the study included 5,935 participants. Figure 1 presents the flowchart of sample selection.

### Variables

This study focused on magnesium intake as the exposure variable. Daily dietary intake data were gathered via 24-h recall interviews and a 30-day dietary supplement questionnaire. For each NHANES 2017–2018 participant, two 24-h recalls were conducted. The first dietary recall was performed in person at the NHANES Mobile Examination Centers (MEC), and the second via telephone by trained interviewers 3–10 days post-MEC interview. The United States Department of Agriculture's Food and Nutrient Database for Dietary Studies was the source of information on nutrient intakes, including dietary fiber (20). The total amount of magnesium consumed per day from food and dietary supplements was determined. The outcome variable, CAP, was measured using the FibroScan^®^ 502 V2 Touch, equipped with liver ultrasonography transient elastography. This device measures CAP by recording ultrasonic attenuation, indicative of hepatic steatosis and liver fat content.

Categorical variables such as gender, race/ethnicity, education level, marital status, smoking habit, diabetes, hypertension, and cholesterol levels were included in our study. Age, body mass index (BMI), γ-glutamyl transpeptidase (GGT), aspartate aminotransferase (AST), alanine aminotransferase (ALT), serum albumin, serum creatinine, and uric acid were among the continuous factors in our study. You may find detailed information about CAP, magnesium intake, and other variables at http://www.cdc.gov/nchs/nhanes/.

### Statistical analysis

We utilized a weighted variance estimation technique to tackle notable fluctuations in our dataset. For categorical data, the weighted chi-square test was utilized to evaluate group differences, and for continuous variables, the weighted linear regression model was employed. The beta values and 95% confidence intervals were calculated using weighted multivariate linear regression analysis between the magnesium intake and CAP. For the purpose of subgroup analysis, stratified multivariate regression analysis was performed, and their interactions were tested. A combination of smooth curve fits and generalized additive models were utilized to investigate the non-linear relationship between CAP and magnesium intake. After non-linearity was identified, we used a recursive method to identify the inflection point in the connection between magnesium intake and CAP, and on either side of this point, we applied a two-piecewise linear regression model. All analyses were conducted using R (http://www.Rproject.org) and EmpowerStats (http://www.empowerstats.com), considering a *P*-value <0.05 as statistically significant.

## Results

Our study comprised 5,935 participants. The population characteristics based on magnesium intake tertiles are shown in Table 1. Higher magnesium intake was associated with an older age, male predominance, non-Hispanic White race, higher education and income levels, and married/partnered marital status. Higher cholesterol levels, liver enzyme levels (AST, ALT, GGT), serum albumin, serum creatinine, uric acid and CAP values increased with higher magnesium intake. No significant differences were seen across tertiles for BMI, smoking rates, diabetes or hypertension prevalence.

**Table 1:** Weighted characteristics of the study population based on magnesium intake tertiles.

**Magnesium intake (mg/day)**	**Total (*N* = 5,935)**	**Low (10.0–185.5, *N* = 1977)**	**Middle (185.6–284.0,p20mm *N* = 1,977)**	**High (284.1–1,097.0, *N* = 1,981)**	***P*-value**
Age (years)	44.83 ± 20.16	40.80 ± 21.08	45.01 ± 20.76	47.75 ± 18.30	<0.0001
Gender (%)					<0.0001
Men	47.51	38.07	43.39	58.40	
Women	52.49	61.93	56.61	41.60	
Race/ethnicity (%)					<0.0001
Mexican American	9.76	9.51	9.52	10.17	
Other Hispanic	7.38	7.76	7.91	6.63	
Non-Hispanic White	62.77	59.67	61.73	66.06	
Non-Hispanic Black	11.22	14.71	11.66	8.16	
Other race	8.86	8.35	9.17	8.99	
Education level (%)					<0.0001
Less than high school	10.41	14.42	9.09	8.93	
High school	28.02	35.70	29.54	21.88	
More than high school	61.57	49.89	61.37	69.19	
Marital status (%)					<0.0001
Married/living with partner	64.06	56.60	62.17	70.35	
Widowed/divorced/separated	18.91	21.58	21.08	15.45	
Never married	17.03	21.83	16.75	14.19	
Income to poverty ratio	3.14 ± 1.63	2.78 ± 1.65	3.13 ± 1.61	3.43 ± 1.59	<0.0001
BMI (kg/m^2^)	29.30 ± 7.49	29.15 ± 7.83	29.55 ± 7.72	29.20 ± 6.98	0.2007
Smoked at least 100 cigarettes in life (%)					0.0544
Yes	38.28	40.03	39.23	36.33	
No	61.72	59.97	60.77	63.67	
Diabetes (%)					0.8733
Yes	10.78	11.14	10.67	10.59	
No	86.82	86.56	86.67	87.16	
Borderline	2.40	2.30	2.66	2.25	
Hypertension (%)					0.4496
Yes	31.80	31.40	32.91	31.13	
No	68.20	68.60	67.09	68.87	
High cholesterol level (%)					<0.0001
Yes	34.12	30.76	32.99	37.42	
No	65.88	69.24	67.01	62.58	
AST (IU/L)	21.06 ± 10.32	20.43 ± 12.14	20.54 ± 8.46	21.96 ± 10.26	<0.0001
ALT (IU/L)	21.62 ± 14.75	20.18 ± 15.34	21.03 ± 14.31	23.19 ± 14.52	<0.0001
GGT (IU/L)	26.36 ± 30.09	25.01 ± 32.21	27.81 ± 32.76	26.11 ± 25.70	0.0263
Serum albumin (g/L)	41.22 ± 3.23	41.02 ± 3.34	41.19 ± 3.30	41.40 ± 3.08	0.0022
Serum creatinine (mg/dL)	0.85 ± 0.30	0.83 ± 0.26	0.84 ± 0.29	0.88 ± 0.32	<0.0001
Uric acid (mg/dL)	5.29 ± 1.38	5.18 ± 1.42	5.25 ± 1.38	5.40 ± 1.33	<0.0001
CAP (dB/m)	261.47 ± 63.66	255.96 ± 63.55	265.24 ± 65.89	262.36 ± 61.41	<0.0001

Mean ± SD for continuous variables: the *P*-value was calculated by the weighted linear regression model. (%) for categorical variables: the *P*-value was calculated by the weighted chi-square test.

Table 2 shows the results of the multivariate linear regression analysis. In the unadjusted Model 1, magnesium intake showed no significant association with CAP (β = 0.01, 95% CI: −0.00, 0.02, *P* = 0.1402). But when age, sex, and race/ethnicity were taken into account, Model 2 showed a significant correlation between increased magnesium intake and decreased CAP (β = −0.02, 95% CI: −0.04, −0.01; *P* = 0.0003). Even after accounting for extra factors, Model 3′s negative connection persisted (β = −0.01, 95% CI: −0.03, −0.00; *P* = 0.0477). Analysis by tertiles of magnesium intake in Model 3 showed that the highest tertile had lower CAP than the lowest tertile (β = −3.60, 95% CI: −7.61, 0.41, *P* = 0.0782), demonstrating a significant linear trend (*P* for trend = 0.036).

**Table 2:** The association between magnesium intake (mg/day) and controlled attenuation parameter (dB/m).

	**Model**	**Model 2**	**Model 3**
	**β (95% CI) *P*-value**	**β (95% CI) *P*-value**	**β (95% CI) *P*-value**
Magnesium intake (mg/day)	0.01 (−0.00, 0.02) 0.1402	−0.02 (−0.04, −0.01) 0.0003	−0.01 (−0.03, −0.00) 0.0477
Magnesium intake tertile			
Low (10.0–185.5 mg/day)	Reference	Reference	Reference
Middle (185.6–284.0 mg/day)	9.28 (5.17, 13.39) <0.0001	4.17 (0.25, 8.10) 0.0373	2.42 (−1.67, 6.50) 0.2462
High (284.1–1,097.0 mg/day)	6.39 (2.39, 10.39) 0.0017	−4.18 (−8.09, −0.27) 0.0364	−3.60 (−7.61, 0.41) 0.0782
*P* for trend	0.004	0.019	0.036

Model 1: no covariates were adjusted.

Model 2: age, gender, and race/ethnicity were adjusted.

Model 3: age, gender, race/ethnicity, education level, marital status, body mass index, smoking behavior, and the existence of diabetes, hypertension, and high cholesterol level, aspartate aminotransferase, alanine aminotransferase, γ- glutamyl transpeptidase, serum albumin, serum creatinine and uric acid were adjusted.

In the subgroup analysis stratified by gender and race/ethnicity, the model is not adjusted for gender and race/ethnicity, respectively.

Table 3 details stratified analyses of the association between magnesium intake and CAP. After adjusting for confounding factors in the study, the inverse link between magnesium intake and CAP remained significant for women (β = −0.02, 95% CI: −0.04, −0.01, *P* = 0.0091), White people (β = −0.02, 95% CI: −0.04, −0.00, *P* = 0.0307) and people without hypertension (β = −0.02, 95% CI: −0.04, −0.01, *P* = 0.0070), and interaction tests further confirmed the significance of the differences between groups (all *P* for interaction <0.05). In gender, education level, marital status, smoking behavior, diabetes, and high cholesterol subgroups, the interaction effect was not statistically significant, indicating that the inverse association between magnesium intake and liver steatosis deposition remains consistent across these subgroups.

**Table 3:** Stratified analyses of the association between magnesium intake (mg/day) and controlled attenuation parameter (dB/m).

**Subgroup**	**β (95% CI) P value**	***P* for interaction**
Age (years)		0.5169
<45	−0.02 (−0.04, 0.00) 0.0765	
45–60	−0.00 (−0.02, 0.02) 0.9278	
>60	−0.01 (−0.04, 0.01) 0.2605	
Gender		0.0389
Men	−0.00 (−0.02, 0.01) 0.7084	
Women	–0.02 (–0.04, –0.01) 0.0091	
Race/ethnicity		0.0495
Mexican American	−0.00 (−0.04, 0.03) 0.9272	
Other Hispanic	0.00 (−0.04, 0.04) 0.9241	
Non-Hispanic White	–0.02 (–0.04, –0.00) 0.0307	
Non-Hispanic Black	0.02 (−0.01, 0.05) 0.1851	
Other Race	0.02 (−0.01, 0.06) 0.1768	
Education level		0.5770
Less than high school	0.01 (−0.04, 0.05) 0.7937	
High school	−0.01 (−0.03, 0.02) 0.5507	
More than high school	–0.02 (–0.03, –0.00) 0.0352	
Marital status		0.3185
Married/living with partner	−0.01 (−0.02, 0.01) 0.3308	
Widowed/ divorced/ separated	–0.03 (–0.06, –0.00) 0.0310	
Never married	−0.02 (−0.04, 0.01) 0.3058	
Smoked at least 100 cigarettes in life		0.0803
Yes	–0.03 (–0.05, –0.01) 0.0130	
No	−0.00 (−0.02, 0.01) 0.7064	
Diabetes		0.0649
Yes	−0.01 (−0.05, 0.03) 0.6527	
No	−0.01 (−0.02, 0.00) 0.1117	
Borderline	–0.10 (–0.18, –0.03) 0.0090	
Hypertension		0.0070
Yes	0.01 (−0.01, 0.04) 0.2381	
No	–0.02 (–0.04, –0.01) 0.0022	
High cholesterol level		0.5610
Yes	−0.01 (−0.03, 0.01) 0.5126	
No	−0.01 (−0.03, 0.00) 0.0689	

Age, gender, race/ethnicity, education level, marital status, body mass index, smoking behavior, and the existence of diabetes, hypertension, and high cholesterol level, aspartate aminotransferase, alanine aminotransferase, γ- glutamyl transpeptidase, serum albumin, serum creatinine, and uric acid were adjusted.

Each stratification adjusted for the above factors except the stratification factor itself.

**Figure 2:**
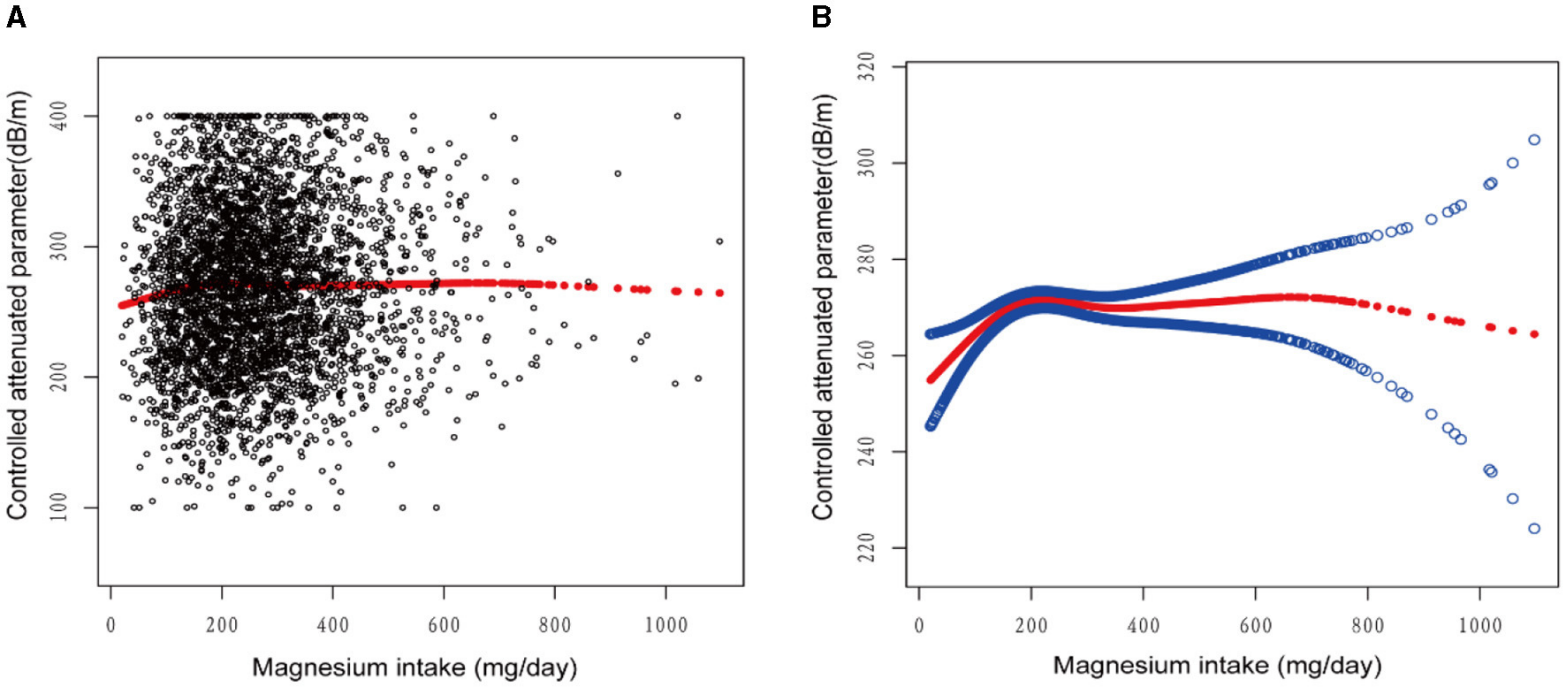
The association between magnesium intake and controlled attenuation parameter. **(A)** Each black point represents a sample. **(B)** Solid rad line represents the smooth curve fit between variables. Blue bands represent the 95% of confidence interval from the fit. Age, gender, race/ethnicity, education level, marital status, body mass index, smoking behavior, and the existence of diabetes, hypertension, and high cholesterol level, aspartate aminotransferase, alanine aminotransferase, γ- glutamyl transpeptidase, serum albumin, serum creatinine, and uric acid were adjusted.

**Figure 3:**
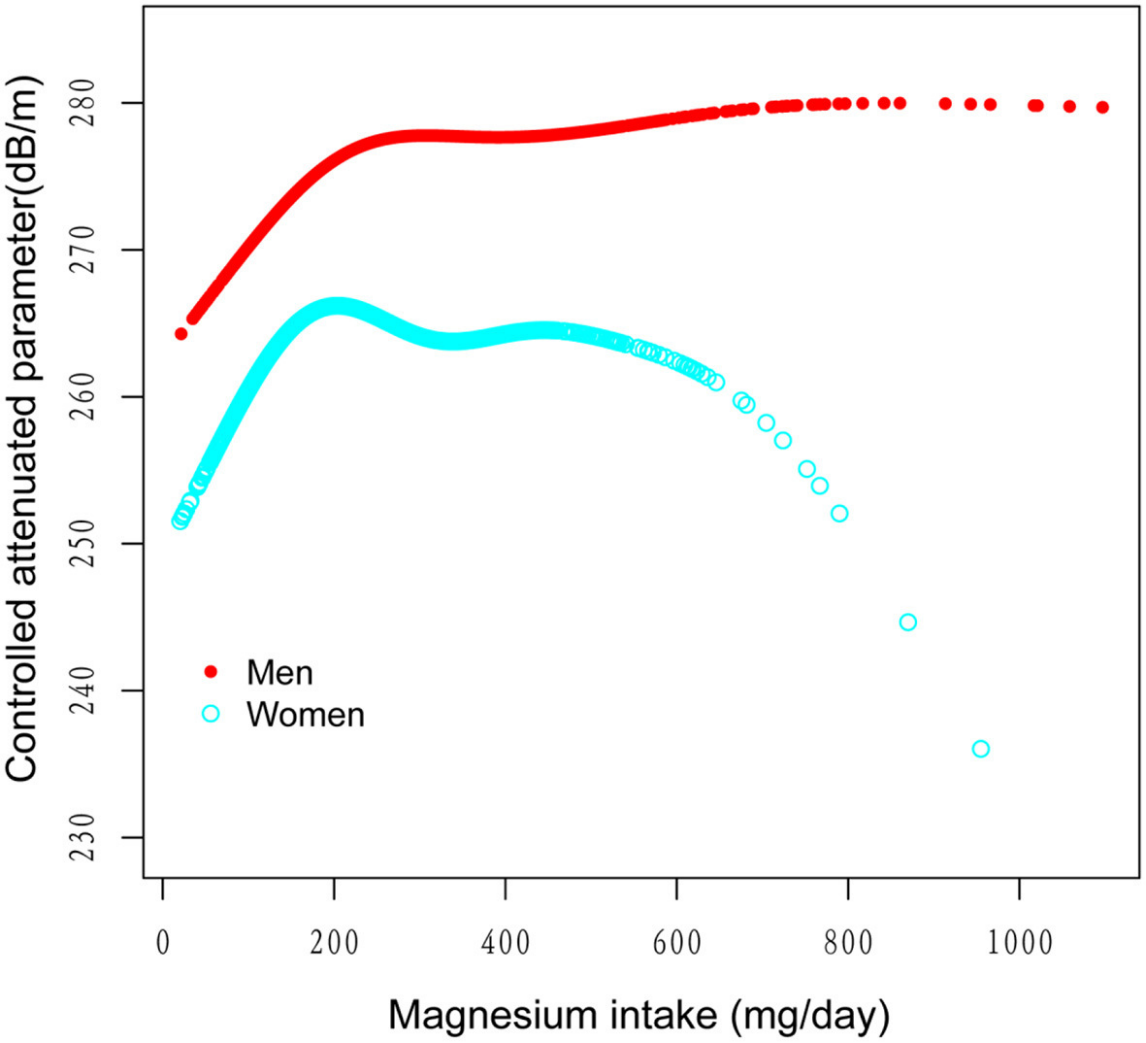
The association between magnesium intake and controlled attenuation parameter stratified by gender. Age, race/ethnicity, education level, marital status, body mass index, smoking behavior, and the existence of diabetes, hypertension, and high cholesterol level, aspartate aminotransferase, alanine aminotransferase, γ- glutamyl transpeptidase, serum albumin, serum creatinine, and uric acid were adjusted.

**Figure 4:**
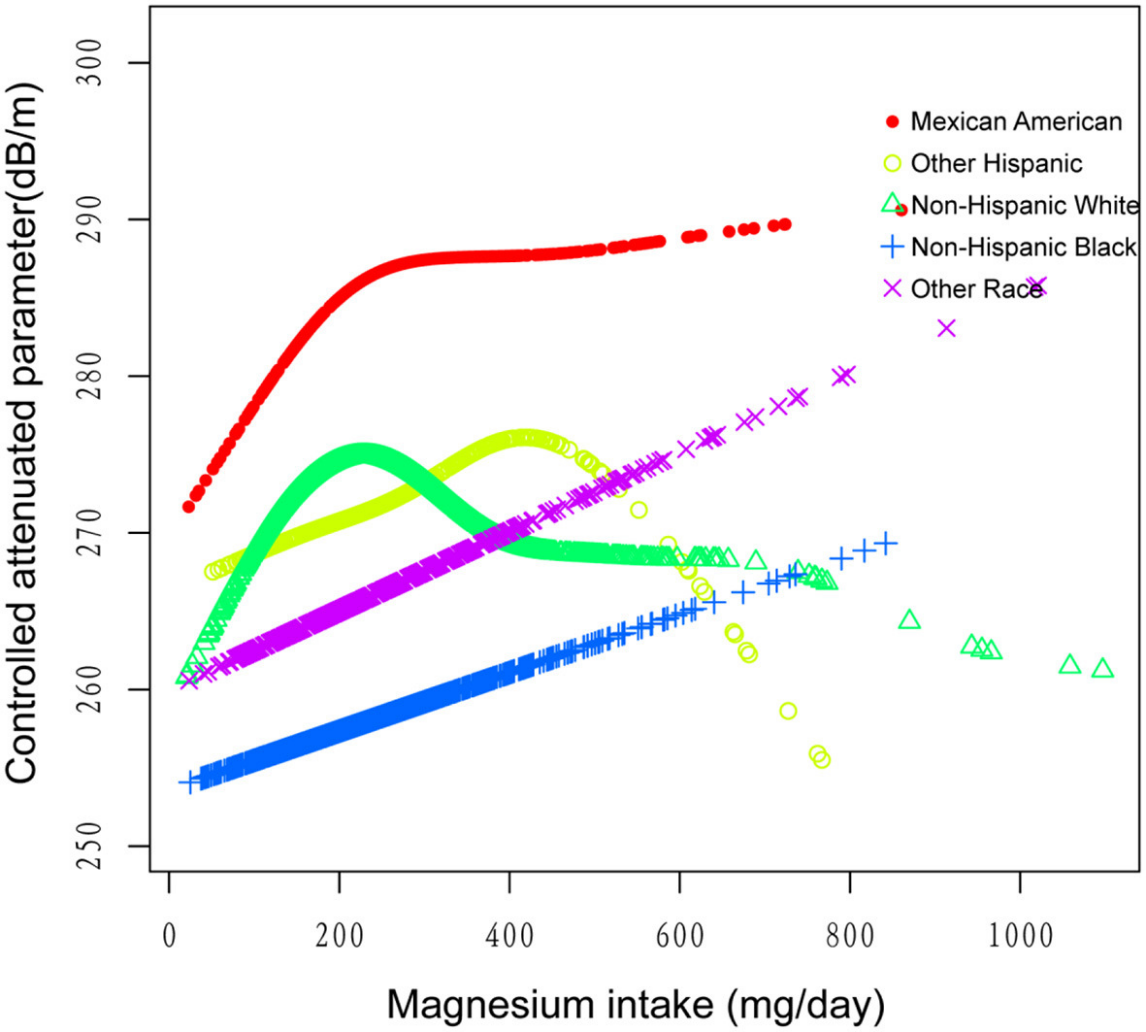
The association between magnesium intake and controlled attenuation parameter stratified by race/ethnicity. Age, gender, education level, marital status, body mass index, smoking behavior, and the existence of diabetes, hypertension, and high cholesterol level, aspartate aminotransferase, alanine aminotransferase, γ- glutamyl transpeptidase, serum albumin, serum creatinine, and uric acid were adjusted.

**Figure 5:**
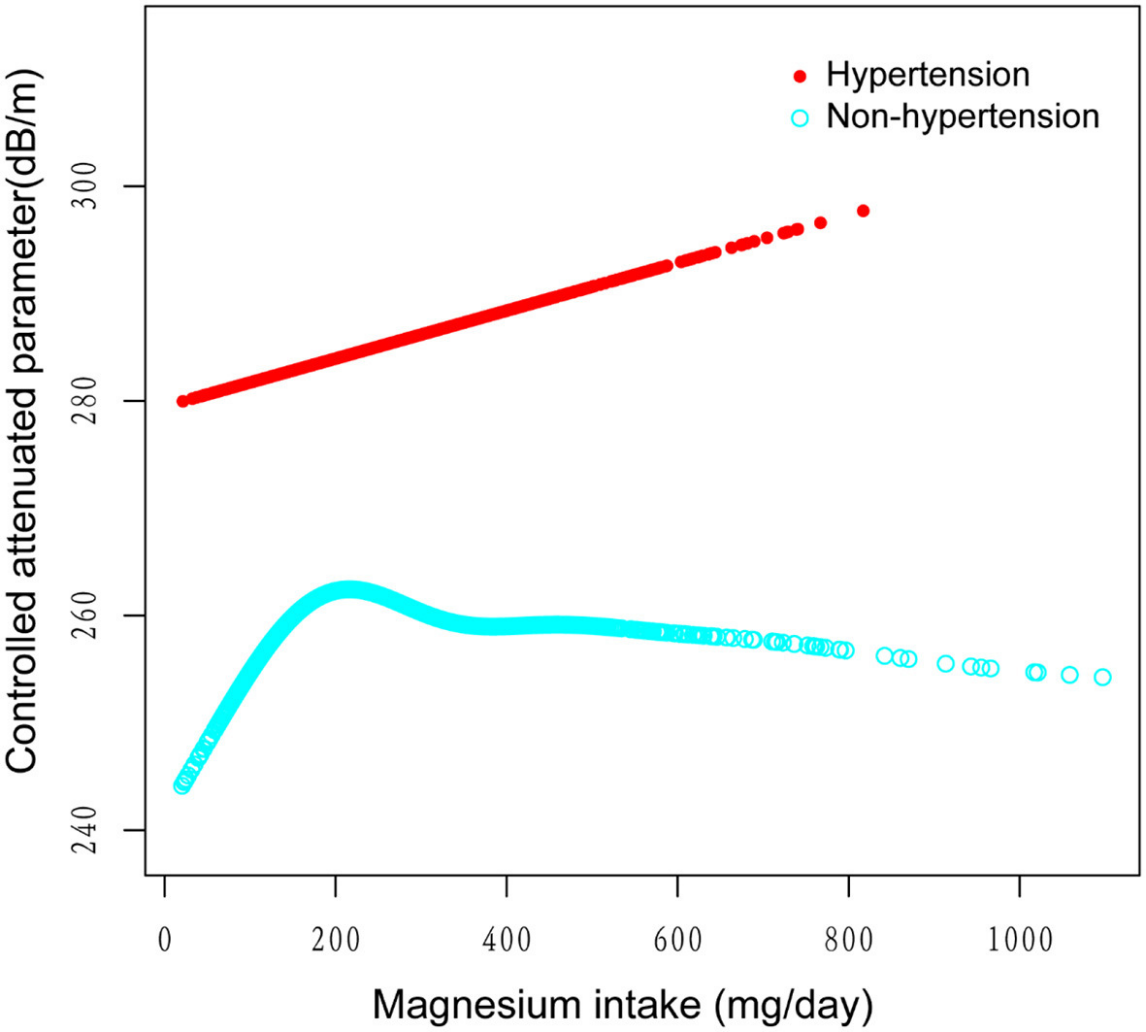
The association between magnesium intake and controlled attenuation parameter stratified by the existence of diabetes. Age, gender, race/ethnicity, education level, marital status, body mass index, smoking behavior, and the existence of diabetes, and high cholesterol level, aspartate aminotransferase, alanine aminotransferase, γ- glutamyl transpeptidase, serum albumin, serum creatinine, and uric acid were adjusted.

Figures 2–5 illustrate smooth curve fits and generalized additive models, demonstrating an inverted *U*-shaped relationship between magnesium intake and CAP in women, White people and people without hypertension. As shown in Table 4, in the female subgroup, the standard linear model showed a significant inverse association between magnesium intake and controlled attenuation parameter (CAP) (β = −0.02, 95%CI: −0.04, −0.01, *P* = 0.0091). The two-piecewise linear regression model revealed a threshold point at 126 mg/day. Below this threshold, there was a significant positive association between magnesium intake and CAP (β = 0.31, 95% CI: 0.13, 0.48, *P* = 0.0005); above the threshold, a significant inverse association was observed (β = −0.04, 95% CI: −0.06, −0.02, *P* = 0.0001). Comparing the goodness of fit between the two-piecewise linear regression models and the standard linear model, the log-likelihood ratio test revealed a significant difference (*P* < 0.001), providing further support for the use of the two-piecewise regression model to capture threshold effects. Similarly, in the non-Hispanic white subgroup and the subgroup without hypertension, comparisons of the goodness of fit between the two-piecewise linear regression models and the standard linear model showed significant differences in the log-likelihood ratio tests (*P* = 0.001 and *P* = 0.004, respectively). In the White people, inflection points occur at a magnesium intake of 124.5 mg/day, as evidenced by a significant likelihood ratio (*P* = 0.001). For magnesium intakes below 124.5 mg/day, each 1 mg/day increase was related to a 0.36 dB/m raise in CAP (95% CI: 0.13, 0.60, *P* = 0.0026); by comparison, for individuals with a magnesium intake >124.5 mg/day, a 1 mg/day upregulation was connected with a 0.03 dB/m drop in CAP (95% CI: −0.05, −0.01, *P* = 0.0022). In the subgroup without hypertension, the two-piecewise linear regression model indicated a threshold value of 125 mg/day. When magnesium intake was below 125 mg/day, each 1 mg/day increase resulted in a 0.25 dB/m increase in CAP (95% CI: 0.07, 0.44, *P* = 0.0077); whereas when magnesium intake exceeded 125 mg/day, each 1 mg/day increase led to a decrease in CAP by 0.03 dB/m (95% CI: −0.04, −0.01, *P* = 0.0003).

**Table 4:** Threshold effect analysis of magnesium intake on controlled attenuation parameter using the two-piecewise linear regression model.

**Controlled attenuation parameter**	**Adjusted β (95% CI), *P*-value**
**Women**	
Fitting by the standard linear model	−0.02 (-0.04,−0.01) 0.0091
**Fitting by the two-piecewise linear model**	
Inflection point	126
Magnesium intake <126 (mg/day)	0.31 (0.13, 0.48) 0.0005
Magnesium intake > 126 (mg/day)	−0.04 (−0.06, −0.02) 0.0001
Log likelihood ratio	<0.001
**Non-Hispanic White**	
Fitting by the standard linear model	−0.02 (−0.04, −0.00) 0.0307
**Fitting by the two-piecewise linear model**	
Inflection point	124.5
Magnesium intake <124.5 (mg/day)	0.36 (0.13, 0.60) 0.0026
Magnesium intake > 124.5 (mg/day)	−0.03 (−0.05, −0.01) 0.0022
Log likelihood ratio	0.001
**Non-hypertension**	
Fitting by the standard linear model	−0.02 (−0.04, −0.01) 0.0048
**Fitting by the two-piecewise linear model**	
Inflection point	125
Magnesium intake <125 (mg/day)	0.25 (0.07, 0.44) 0.0077
Magnesium intake > 125 (mg/day)	−0.03 (−0.04, −0.01) 0.0003
Log likelihood ratio	0.004

Age, gender, race/ethnicity, education level, marital status, body mass index, smoking behavior, and the existence of diabetes, hypertension, and high cholesterol level, aspartate aminotransferase, alanine aminotransferase, γ- glutamyl transpeptidase, serum albumin, serum creatinine, and uric acid were adjusted.

Each stratification adjusted for the above factors except the stratification factor itself.

## Discussion

This study analyzed 2017–2020 NHANES data from the United States to explore the relationship between magnesium intake and liver fat accumulation, using CAP values as a measure. The multivariate regression analysis indicated a trend of decreasing CAP values correlating with increased magnesium intake. This inverse relationship between magnesium intake and hepatic steatosis is consistent with prior research. A study of 226 healthy individuals demonstrated an independent association between lower serum magnesium levels and biopsy-confirmed hepatic steatosis (13). Another analysis of the NHANES III cohort indicated a potential link between higher magnesium intake and lower liver disease mortality in hepatic steatosis patients (21). Furthermore, a study in which mice were fed a magnesium-deficient diet revealed increased levels of hepatic steatosis, swelling, and overall scores in comparison to a control group (22).

In a study on metabolic dysfunction-associated steatotic liver disease in the Korean NHANES database (23), magnesium intake was not mentioned in relation to MASLD, possibly due to variations in study populations and definitions of fatty liver. Our study, on the other hand, accounted for a broader range of confounding factors, potentially offering a more in-depth exploration of the true relationship between the two variables.

Magnesium deficiency is associated with an increased risk of liver steatosis through several pathways. It disrupts fatty acid and triglyceride metabolism, leading to fat accumulation in the liver (22). The deficiency also interferes with insulin signaling and exacerbates inflammation and oxidative stress in the liver (24). Additionally, magnesium deficiency may increase liver adipocytes that produce proteases enhancing angiotensin II, further worsening steatosis (25). Conversely, magnesium supplementation can regulate fatty acid metabolism, promote fatty acid oxidation, and activate the AMP-activated protein kinase-mammalian rapamycin target protein (AMPK-mTOR) pathway to induce autophagy in liver cells. This helps reduce intracellular fat deposition and thereby prevent or improve hepatic steatosis (22).

Our subgroup analysis revealed significant non-linear associations between magnesium intake and CAP in the female, White people, and non-hypertension subgroups, each showing a distinct turning point. Below this point, magnesium intake and CAP were positively correlated, while above it, they exhibited a significant inverse relationship. These findings suggest that the optimal level of magnesium intake may vary by gender, ethnicity, and hypertension status. To our knowledge, this may be the first study to unveil the complex non-linear relationship between magnesium intake and liver fat accumulation, indicating potential gender, ethnic, and hypertension-related differences. These variations may be attributable to genetic risk factors, lifestyle differences, and other elements (26). Further research, involving larger sample sizes and a prospective approach, is necessary for validation. The scale of our study, using data from the NHANES cohort, strengthens our findings, given NHANES' nationally representative scope. This discovery holds significant clinical implications by emphasizing the need to consider individual characteristics when assessing the role of magnesium in preventing and managing hepatic steatosis. Nevertheless, our study has limitations; as a cross-sectional study, it establishes only a correlation, not a causal relationship, between magnesium intake and liver fat. Additionally, the magnesium intake data, based on two 24-h dietary recalls, could be influenced by reporting biases. Moreover, as our sample exclusively comprises U.S. participants, this might limit the generalizability of our findings to international populations. Lastly, there may be other potential biases stemming from additional confounding factors. For instance, due to limitations in the NHANES database, not all cardiac metabolic factors are available, leading to an incomplete assessment of cardiac metabolic risk factors.

## Conclusions

Our research suggests an inverse relationship between magnesium intake and hepatic steatosis in the majority of Americans. In women, whites, and non-hypertensive individuals, the relationship followed an inverted U-curve, with turning points at 126, 124.5, and 125 mg/day, respectively. The impact of magnesium intake on CAP values varies before and after these turning points. These findings inform clinical nutritional interventions and personalized magnesium intake, underscoring the significance of magnesium research in developing pharmacological approaches to reverse liver steatosis.

## Data availability statement

Publicly available datasets were analyzed in this study. This data can be found here: https://www.cdc.gov/nchs/nhanes/index.htm.

## Ethics statement

The studies involving humans were approved by National Center for Health Statistics Research Ethics Review Board. The studies were conducted in accordance with the local legislation and institutional requirements. Written informed consent for participation in this study was provided by the participants' legal guardians/next of kin.

## Author contributions

XC: Data curation, Investigation, Methodology, Software, Writing – original draft. LF: Investigation, Writing – review & editing. ZZ: Project administration, Writing – review & editing. YW: Methodology, Project administration, Writing – original draft, Writing – review & editing.
